# Near-infrared fluorescence imaging for ureteral and sentinel lymph node mapping in gynecological minimally invasive surgery: a review of fluorophores and domestic laparoscopic systems

**DOI:** 10.1186/s12957-026-04516-y

**Published:** 2026-08-19

**Authors:** Tongxin Chen, Haili Li

**Affiliations:** Department of Gynecology, The Second Clinical Medical College of Jinan University, Shenzhen People’s Hospital, Shenzhen, Guangdong 518020 China

## Abstract

As laparoscopic techniques continue to advance, intraoperative tissue visualization has become increasingly important. In gynecological oncology, sentinel lymph node (SLN) mapping remains a critical procedure for assessing lymph node metastasis and guiding lymphadenectomy. Meanwhile, iatrogenic ureteral injury, although rare, can have severe consequences. Thus, achieving rapid, reliable, and cost-effective intraoperative visualization of both SLNs and the ureters during laparoscopy poses a significant clinical challenge. Near-infrared fluorescence (NIRF) imaging has emerged as a promising solution, using various fluorophores such as methylene blue (MB), indocyanine green (ICG), and technetium-99m (^99m^Tc) to enable clear visualization of living tissues. However, a comprehensive summary of the advantages and limitations of these fluorophores is lacking. This article reviews the efficacy of different fluorophores for SLN and ureteral mapping and discusses recent advances in domestic fluorescence laparoscopic systems.

## Introduction to NIRF

In recent years, fluorescence-guided surgery (FGS) has become a promising new technology. Combined with traditional open surgery, laparoscopy, and emerging robotic surgery, it enhances surgical precision and safety. Near-infrared imaging is a novel intraoperative in vivo imaging modality within this field.

### Components and principles of NIRF

NIRF technology consists of fluorescent probes and an imaging system. The principle involves injecting a fluorescent probe (fluorophore) into tissue, illuminating the area with near-infrared light, and capturing the fluorescence emitted by the target tissue. This signal is detected by a highly sensitive camera and processed to produce anatomical localization images [1].

### Advantages of NIRF

Near-infrared (NIR) light, the first non-visible region discovered in the absorption spectrum [2], enables active fluorescence signal generation. Fluorophores (typically ICG) are excited by NIR light at approximately 780 nm, and their subsequent relaxation to the ground state results in light emission at a higher wavelength (approximately 820 nm) [3].Compared with traditional molecular imaging, optical imaging in the NIR spectrum (λ > 650 nm) offers four key advantages: increased tissue penetration, reduced scattering, a higher signal-to-background ratio (SBR), and lack of radioactivity [4]. Compared to light in the visible spectrum (400–650 nm), NIR light minimizes absorption by physiologically abundant molecules like hemoglobin and lipids, thereby increasing penetration depth and achieving higher image contrast [5]. Additionally, autofluorescence intensity is significantly lower within the NIR spectrum. The detection systems for optical fluorescence imaging are relatively inexpensive and portable, and when combined with its minimally invasive nature, make it a highly promising bedside diagnostic technology [1]. Biological tissues have relatively low absorption and scattering in the NIR range (700–900 nm), allowing photons to penetrate relatively deeply. By introducing exogenous NIR fluorophores into the surgical field, desired targets such as the ureter can be illuminated with high sensitivity and specificity [6].

Currently, MB and ICG are the most commonly used fluorophores in clinical practice and play a vital role in intraoperative real‑time NIR fluorescence imaging. However, most current clinical imaging systems can only capture a single fluorescence wavelength at a time and cannot simultaneously visualize multiple fluorophores. For example, during surgery for gynecologic malignancies, when pelvic lymph nodes need to be dissected, it is also essential to visualize the ureter to avoid iatrogenic injury. With conventional systems, surgeons must repeatedly switch between fluorescence views to locate both structures, and this process often lacks precision. This review summarizes the fluorescent dyes currently used in clinical practice to facilitate further research in fluorescence imaging. In addition, it highlights newly developed fluorescence imaging devices in China, with a particular focus on the dual‑fluorescence imaging system, which can simultaneously display two fluorescence signals on a single monitor and integrate them with white‑light images for more accurate intraoperative localization.

## Fluorescent dye probes

Common NIRF tracers include bioactive dyes, radionuclides, and fluorescent dyes such as ICG and MB. An ideal NIRF tracer should exhibit minimal interference from biological samples, low fluorescence scattering, and strong tissue penetration. Administration routes mainly include intravenous and tissue injection. For SLN mapping, tissue injection is typically used; for ureteral imaging, intravenous or intraureteral injection is preferred to reduce the risk of ureteral injury and shorten operative time.

### Radionuclides

^99m^Tc is a diagnostic radionuclide. After administration, tissue absorption is low. Its chemical and biological properties resemble iodine. ^99m^Tc can serve as an imaging agent or tracer accumulating in specific tissues (e.g., lymph nodes), detected via emitted γ-rays. As ^99m^Tc can display excretion and perfusion, it is commonly used for imaging kidneys, ureters, and tumors. However, the clinical translation of ^99m^Tc-based radiopharmaceuticals faces challenges, necessitating ongoing development of novel, efficient, and simple labeling methods [7].

### Fluorescent dyes

#### ICG

ICG is a water-soluble, anionic, amphiphilic tricarbocyanine probe with a hydrodynamic diameter of 1.2 nm. Its excitation and emission wavelengths in serum are 778 nm and 830 nm, respectively. Approved by the FDA, ICG is excreted via kidneys and liver (primarily liver via bile). Clinically used for over 30 years in ocular angiography, cardiac output measurement, and liver function assessment, ICG fluorescence in NIRF is most commonly applied in: (1) SLN detection; (2) edema assessment; (3) evaluating microvascular circulation in free flaps during reconstructive surgery, with studies indicating good safety [8]. Attempts to conjugate ICG with monoclonal antibodies for targeted tumor localization and therapy exist. However, ICG’s protein-binding capacity can significantly reduce fluorescence intensity upon conjugation [8], hindering NIRF observation. ICG has a high “hydrophobiccharacter”; intravenous administration leads to rapid hepatic uptake, reducing plasma concentration. Since renal excretion is not its primary route, the ICG concentration excreted via ureters further decreases, while hepatic uptake increases fluorescence in bile and gastrointestinal vessels, potentially interfering with fluorescence guidance in abdominal surgery [9]. Therefore, ICG is not routinely administered intravenously but via direct tissue or intracavitary injection (e.g., into lymph node tissue or retrograde via ureteral catheter).

#### Methylene Blue (MB)

MB is another FDA-approved probe with NIR properties. It strongly absorbs broad red light (550–700 nm, peak at 664 nm) and emits fluorescence in the NIR region. When diluted to near-invisibility, MB becomes a medium-intensity fluorophore emitting NIR fluorescence around λ ≈ 700 nm [10]. The maximum penetration depth of MB’s NIR fluorescence imaging is approximately 3–5 mm, effectively penetrating thin adipose tissue to visualize underlying structures [10]. However, MB can cause serious adverse effects such as methemoglobinemia, allergic reactions, interference with pulse oximetry (SpO₂) [3], transient blue urine, and skin discoloration.

#### CW800-CA

CW800-CA is another heptamethine indocyanine fluorophore excreted in urine, with minimal biliary excretion [6]. It fluoresces at wavelengths similar to ICG (700–830 nm) and is detectable by commercially available fluorescence imaging devices. However, CW800-CA lacks FDA approval for clinical use. Like ICG, it belongs to the heptamethine indocyanine class with low inherent toxicity [11]. Differences among heptamethine indocyanines relate to sulfonation degree (hydrophilicity). CW800-CA provides bright ureteral visualization via renal clearance. Feasibility studies in pigs show rapid ureteral visualization after intravenous injection [12].

#### Others

Pudexacianinium (ASP 5354): An ICG derivative with high hydrophilicity. After IV administration, it is rapidly cleared unchanged in urine, designed for accurate, real-time ureteral visualization during surgery. Its optical properties resemble ICG (absorption peak ~ 780 nm, emission ~ 820 nm). Studies show ASP5354 has an excellent SBR, with fluorescence intensity three times that of ICG. Due to its hydrophilic nature, it visualizes in urine upon excitation after IV injection. Doses up to 24 mg are safe and well-tolerated, with 77–100% excreted unchanged [12, 13].

IRDye 800 BK: A fluorophore excreted via liver and kidneys (primarily kidneys) after IV injection. Its maximum absorption (774 nm) and emission (790 nm) wavelengths make it advantageous for NIRF ureteral imaging [14].

ZW800-1: A heptamethine cyanine dye, a zwitterion with high hydrophilicity. Peak absorption at 770 nm and emission ~ 800 nm enable relatively deep penetration and effective imaging of deep tissues. Unlike ICG, ZW800-1 is rapidly cleared by the kidneys after IV injection, exhibits low background binding to normal tissues/organs, and avoids fluorescent contamination of other organs. However, research on ZW800-1 is incomplete, requiring further studies on safety and stability [9, 15].

UL-766: A heptamethine cyanine-based fluorophore with absorption/emission maxima around 800 nm. It shows excellent chemical stability, ease of synthesis, high selectivity, and rapid renal clearance, making it a promising candidate for ureteral marking in fluorescence-guided laparoscopy. Compared to IRDye-800 CW, UL-766 offers almost complete renal clearance and high sensitivity, requiring low doses for strong fluorescence. Its biocompatibility is under investigation, showing potential as an ICG alternative [16].

Pafolacianine (OTL-38): Composed of a folate analog conjugated to the NIR cyanine dye S0456. FDA-approved in 2021 as an optical imaging agent [17], it binds to folate receptor-α (FRα), serving as an excellent NIR fluorophore (excitation max 774–776 nm, emission max 794–796 nm). FRα is highly expressed in many malignant tissues (e.g., breast, ovarian, cervical, lung, brain, kidney), with most (> 90%) FRα-overexpressing cancers being epithelial in origin [18, 19]. Pafolacianine shows unique advantages in ovarian tumor imaging but has limitations such as high false-positive rates [20], complex chemistry, and suboptimal optical properties [21]. Currently in phase III trials [20], it holds promise as a future mainstream fluorescent agent for epithelial tumors.

In gynecology, NIRF with fluorescent dyes is primarily applied for SLN and ureteral mapping, which will be elaborated in the following sections (Table 1).

**Table 1:** Summary of dyes

Full Name	Abbreviation	Dosage	Imaging Time	Advantages	Disadvantages
Indocyanine Green	ICG	0.25 mg/kg (IV)	Visualizes within minutes post- injection; signal persists until end of surgery	High safety profile; FDA- approved; can be administer -ed via retrograde or tissue injection	Reduced visualization in obese patients; prone to hepatic uptake; fluorescence affected by protein binding; penetration depth ~ 5 mm
Methylene Blue	MB	0.75–1.0 mg/kg (IV)	Visualizes 10 min post- injection; lasts 60–75 min	Penetration depth 3–5 mm; low cost; can penetrate superficial adipose tissue	Potential allergic reactions; risk of methemo globinemia; interferes with pulse oximetry; causes urine and skin discoloration
CW800-CA	CW800-CA	0.15 mg/kg (commonly used in animal studies)	Visualizes 5 min post- injection; persists until end of surgery	Rapid renal clearance; high fluores -cence brightness; low toxicity	Not FDA-approved; limited human studies
Pudexacianinium	ASP 5354	1.0–3.0 mg (IV)	Dose-dependent; optimal imag -ing effect at 3.0 mg dose	Fluorescence intensity 3× higher than ICG; rapid renal clearance; almost entirely excreted unchanged	Not yet widely clinically applied; risk of dose-dependent adverse reactions
IRDye 800 BK	IRDye 800	0.15 mg/kg (animal studies)	Visualizes 5 min post -injection; persists until end of surgery	Predominantly renal excretion; stable fluorescence; strong tissue penetration	Not FDA-approved; limited human studies
ZW800−1	ZW800−1	Dosage under investigation (research phase)	Rapid visualization; good pene -tration depth	Rapid renal clearance; low background fluorescence; strong tissue penetration	Safety not fully validated; insufficient clinical research
UL−766	UL−766	Low-dose effective (exact dosage unspecified)	Rapid visualization; high fluore -scence intensity	High renal clearance rate; good chemical stability; high sensitivity	Insufficient toxicity data; not commercially available
Pafolacianine	OTL−38	Dosage varies per clinical trial protocol	Rapid visualization post-injection; persists until end of surgery	Targets folate receptor-α; FDA-approved for ovarian cancer imaging	High false-positive rate; complex chemical composition; suboptimal optical properties

## Sentinel lymph node mapping

The SLN is the first lymph node group receiving lymphatic drainage from an organ or tissue, initially affected by primary malignant metastasis, indicating regional involvement via drainage pathways [22]. SLN biopsy allows timely detection of regional metastasis, enabling avoidance of systematic lymphadenectomy in node-negative patients, reducing trauma and complications, improving postoperative quality of life, and aligning with “minimally invasive” and “precise” surgical concepts. Sentinel node procedure (SNP) was first described by Gould et al. in the 1960s. Standard SNP involves peritumoral injection of radioactive colloids (e.g., ^99m^Tc) and/or MB if radioactive colloids are unavailable or optical guidance is needed. Nodes with radioactivity and/or blue staining are removed and assessed for metastatic cells. The ability of ^99m^Tc and/or MB to guide surgeons to the SLN is the detection rate, independent of metastasis presence [10].

Current SLN detection methods include MB, ICG, radionuclide tracing, and activated carbon tracing.

MB Method (Biological Dye Tracing): Commonly uses MB or isosulfan blue. MB is the most common tracer in China, being simple, low-cost, and effective, requiring no extra imaging equipment for SLN identification in both open and laparoscopic surgery. However, due to its low molecular weight, MB flows rapidly in lymphatics, resulting in a short detection window, difficulty in precise SLN detection, and easy passage to echelon nodes, increasing false-positive rates. Removing echelon nodes does not improve detection but increases postoperative complications. Also, weak protein binding allows some dye to enter capillaries, turning the surgical field blue and hindering identification. Clinically, MB can be diluted to near-invisibility, becoming a medium-intensity fluorophore visible under NIR imaging. The disadvantages of blue dyes are also evident. For example, MB can cause adverse reactions ranging from skin reactions and injection site necrosis to life‑threatening anaphylaxis and hemolytic anemia. In addition, blue dyes have drawbacks such as persistent skin staining, surgical field contamination, and interference with pulse oximetry [3]. Because MB does not bind to albumin, its fluorescence penetration depth is lower than that of ICG, which exhibits a relatively high tissue penetration depth (5–10 mm) [3]. In patients with high body mass index (BMI), NIR imaging can penetrate thicker adipose layers, enabling visualization of sentinel lymph nodes SLNs covered by fat.

ICG Method: Combines ICG with NIR imaging for real-time lymphatic mapping and visual guidance. In gynecologic practice, sentinel lymph node mapping for endometrial cancer is now a standard procedure, with ICG injection commonly performed via the four‑site or nine‑site cervical approach. Salih Taşkın et al. reported a total SLN detection rate of 95.7% (unilateral 18.3%, bilateral 77.4%) with a false-negative rate of 1.56% in 71 early endometrial cancer patients using NIR/ICG laparoscopy [23]. Among these, isolated para‑aortic lymph node metastasis was the sole factor contributing to false‑negative results in this study. This finding is consistent with the results reported by Andrea Papadia et al. [24] in a study on sentinel lymph node (SLN) mapping for endometrial cancer, in which most included patients had early‑stage disease. The false‑negative rate for SLN mapping was 8.3%, all attributable to isolated para‑aortic lymph node metastases. In a study by Emma C. Rossi et al. [25] involving 340 patients with endometrial cancer who underwent SLN biopsy, the sensitivity of SLN detection was 97.2% (95% CI, 85.0%–100%), and the negative predictive value was 99.6% (95% CI, 97.9%–100%). The false‑negative rate was 0.4%, attributed to a single case of isolated para‑aortic lymph node metastasis. However, this patient was excluded from the analysis because she did not meet the inclusion criteria, having previously undergone retroperitoneal mass surgery. Adverse events occurred in 33 of 356 patients (9%), among whom 22 experienced serious adverse events. The most common grade 3 or 4 adverse events or serious adverse events included postoperative neurological changes, such as peripheral nerve injury, or central nervous system symptoms, such as syncope or dizziness (4 cases); postoperative respiratory distress or failure (4 cases); postoperative nausea and vomiting (3 cases); and bowel injury (3 cases).Compared to visual MB observation, ICG uses non-ionizing radiation, shows less diffusion, provides a clearer surgical field, and has no significant side effects, unlike MB [26]. A meta-analysis by Jeremiasse B et al. found ICG superior to MB for unilateral and bilateral SLN detection in breast, gynecologic, and skin cancers [26]. Compared to lymphoscintigraphy with ^99m^Tc, ICG requires no preoperative intervention, used only intraoperatively as needed.

Radionuclide Tracing: Most commonly uses ^99m^Tc-colloid, requiring gamma cameras and SPECT/CT. ^99m^Tc’s main advantage is high penetration through thick fat, suiting high-BMI patients. One study reported an 84% total SLN detection rate using ^99m^Tc alone in 35 ovarian cancer patients [27]. Jeremiasse B et al. found no significant difference in detection rates between ICG and ^99m^Tc for gynecological cancers [26]. Radiation concern exists, but the dose for SLN mapping (2–3 mSv) is far below safety limits (chest CT 7 mSv; symptoms appear at > 1000 mSv) [28]. Kleppe M et al. reported no adverse events in 22 ovarian cancer patients injected with ^99m^Tc [29].

Activated Carbon Tracing: An SLN tracing method with unclear efficacy. CH40 (activated carbon particles), a bioactive dye with good tropism, was previously used for SLN detection. Via macrophage phagocytosis, it enters lymphatics and capillaries, residing in nodes, staining them black for easy identification. CH40 has a long half-life, staying in nodes for up to three days. Its precise clinical efficacy for intraoperative SLN tracing remains unclear, limiting its use.

Given each method’s pros and cons, combined tracers have been developed, offering varying advantages in detection rates (unilateral/bilateral), false-negative rates, and safety.

ICG and ^99m^Tc: ICG’s small size allows rapid lymphatic spread and entry into second-echelon nodes, explaining high misidentification rates. To reduce this, Jeremiasse B’s meta-analysis found that combining ICG with radioactive colloids via non-covalent interactions increases molecular size [26]. Their advantages complement: ^99m^Tc has high penetration but low spatial resolution; ICG has high spatial resolution but lacks deep penetration, performing poorly in high-BMI patients. Combination yields high penetration and resolution. However, research is early-stage, needing more studies on feasibility and advantages [30]. Combining ICG with ^99m^Tc-nanocolloid requires optimal dosing for maximum fluorescence quantum yield and brightness, yet detailed studies are lacking. Vermersch C et al., in 100 node-negative breast cancer patients, compared ICG+^99m^Tc vs. ^99m^Tc alone: total lymphatic vessel detection 88%; unilateral SLN detection 44.0% vs. 40.8% [31]. In 80 endometrial cancer patients, ICG+^99m^Tc yielded unilateral/bilateral detection rates of 97.5% and 81.3% [32]. Combination reduces false-negative rates, crucial during initial training [31]. However, adverse events (allergic reactions, hematoma, pain) occurred in 36.0% of the combination group and 26.5% of the ^99m^Tc-alone group, related to ^99m^Tc [31].

ICG and MB: Rarely reported due to different fluorescence conditions and MB’s significant adverse effects versus ICG’s minimal ones. Robert W Holloway et al. found ICG superior to isosulfan blue for bilateral SLN detection and metastasis identification, but combination achieved 100% bilateral detection [32]. However, mixing ICG with MB did not significantly improve total SLN detection versus ICG alone and introduced adverse effects absent with ICG alone, resulting in limited studies [32].

MB and ^99m^Tc: ^99m^Tc is often combined with MB to improve true-positive rates for unilateral (70–100%) and bilateral (90%) detection [33]. John P. Diaz, M.D. et al. reported a 95% success rate (unilateral and bilateral) using MB and ^99m^Tc for SLN mapping in early cervical cancer [34]. In a study of 48 vulvar cancer patients, MB+^99m^Tc vs. ICG+^99m^Tc identified 52 vs. 41 SLNs. For unilateral tumors, unilateral/bilateral detection rates were 41.7%/20.8% with MB+^99m^Tc; for midline tumors, 4.2%/33.3% [35]. A 342-patient study in early endometrial cancer found no significant difference in total SLN detection between ICG (96.9%) and ^99m^Tc+MB (97.35%), but ICG had better bilateral detection, possibly due to its smaller size [36] (Table 2).

**Table 2:** Summary table of sentinel lymph node mapping methods

Method	Dosage	Imaging Time	Advantages	Disadvantages
MB	Tissue injection, commonly used with diluent	Rapid imaging, but short duration	Low cost, no special equipment required, suitable for open and laparoscopic surgery	Easy diffusion, high false positive rate, may cause allergies, skin staining
ICG	Tissue injection, typically 0.5−1.0 ml	Real-time imaging, lasts until end of surgery	Clear fluorescence, minimal diffusion, high safety profile, high detection rate	Limited penetration depth in obese patients
Radioisotope Tracer	Preoperative injection, dose approx. 2–3 mSv	Preoperative imaging possible, intraoperative detection	Strong penetration, suitable for high BMI patients, high detection rate	Requires radioactive equipment, radiation risk, requires preoperative preparation
ICG + ^99m^Tc Combined	Tissue injection combined with preoperative radioactive injection	Real-time imaging + Preoperative localization	High penetration + High resolution, higher detection rate, low false negative rate	Requires two types of equipment, complex procedure, may increase adverse reactions
ICG + MB Combined	Combined tissue injection	Real-time imaging	Detection rate can reach 100%, complementary visualization	Increased risk of adverse reactions, limited studies
MB + ^99m^Tc Combined	Tissue injection + Preoperative radioactive injection	Real-time + Preoperative imaging	High detection rate, especially suitable for vulvar cancer, etc.	Requires two methods, complex procedure, potential staining and radiation risks

* *ICG* Indocyanine Green, *MB* Methylene Blue, ^99m^*Tc* Technetium-99m

## Ureteral mapping

Over the past 30 years, laparoscopic surgery has flourished across specialties, including gynecology, offering shorter recovery, less pain, reduced analgesic use, less blood loss, and lower wound infection rates, making it the preferred approach for most gynecological oncology surgeries [37]. Endometrial cancer was the first gynecological malignancy treated laparoscopically, and since the 21st century, laparoscopy has become the gold standard for its surgical treatment [38].

Despite technological advances, urological complications remain a major concern in gynecological surgery, with half of iatrogenic ureteral injuries originating from gynecological procedures. Urological complications include intraoperative and postoperative bladder (injury, retention, incontinence, fistula) and ureteral (injury, obstruction, fistula) complications [37]. In a single-center prospective cohort study, Vito Andrea Capozzi et al. [37] found 9 urological complications among 260 laparoscopic surgeries (including endometrial cancer, uterine sarcoma, cervical cancer, ovarian cancer, borderline ovarian cancer). Hwang et al. [39] meta-analysis reported a 6.0% intra/postoperative urological complication rate for radical hysterectomy, nearly double that of laparotomy. This section focuses on ureteral injury.

Risk factors for ureteral injury in laparoscopy include prior surgery/C-section, adhesions from tumors, endometriosis, large fibroids, adnexal masses, or prior urogynecologic surgery distorting the ureteral course [37]. Common injury sites: pelvic brim, common iliac artery bifurcation, lateral pelvic wall, uterine artery crossing, and ureterovesical junction [40]. To reduce risk, Stefano Uccella et al. [41] suggested ureteral rolling in advanced cancer patients may offer some protection. However, this may damage ureteral capillaries/arterioles, reduce blood supply increasing fistula risk, and damage the ureteral sheath/mesentery, leading to hypogastric nerve injury, ureteral dilation, and detrusor hyperactivity [42]. Prophylactic ureteral stent placement can reduce injury, shorten operative time, and reduce blood loss, serving as a routine preoperative option, especially in surgeries with pelvic adhesions [43]. A retrospective study of 146 prophylactic bilateral stent placements reported only 2 ureteral injuries [34]. Preoperative stenting may reduce intraoperative injury rates, but is debated; M O Schimpf [44] suggested bilateral stenting is cost-effective only if the ureteral injury rate exceeds 3.2% in total hysterectomy .

Iatrogenic ureteral injury may relate to preoperative ureteral catheterization, stent placement, or intraoperative cystoscopy. While intravenous/retrograde pyelography and CT urography can reduce injury probability, they lack real-time visual guidance [12]. NIRF provides real-time fluorescence imaging, clearly marking the ureter intraoperatively, making it stand out. Currently, the most commonly used fluorophores are MB and ICG.

The first human trial of MB for ureteral imaging was in 2013 by Verbeek et al. [45], who intravenously injected MB and used NIRF for ureteral visualization, successfully imaging bilateral ureters in 12 patients within 10 min. Multiple studies report high success rates with MB [46, 47]. Mahdi Al-Taher, in 12 colorectal cancer surgeries, used IV MB (0.125-0.50 mg/kg initially) with NIRF, achieving weak fluorescence in only 2 of 8 patients. Increasing the dose to 0.75-1.0 mg/kg resulted in clear ureteral visualization in 2 patients. They [48] also measured fluorescence intensity in ex vivo urine at different dilutions, finding SBR decreased at both low and high dilutions, with an optimal mid-range, guiding optimal urine MB concentration. Addressing small sample bias, Barnes T. G. (2018) [49]studied 40 laparoscopic colorectal cancer patients (69 ureters) using IV MB at 0.25, 0.5, 0.75, and 1 mg/kg doses, achieving a 91.3% (63/69) success rate, with visualization as early as 10 min post-injection. Another [50] advantage is MB’s visualization duration of 60–75 min, enhancing safety. However, MB’s NIRF penetration is only 3–5 mm, potentially limiting visualization in high-BMI patients. IV MB can cause adverse effects like intraoperative SpO₂ reduction (to as low as 95%, reversible with oxygen), green urine postoperatively [51], and other postoperative issues (abdominal pain, bloating, allergy, dizziness, headache, nausea, vomiting, cardiovascular symptoms) [51]. Due to serotonin syndrome risk, MB should not be used in patients on SSRIs.

In summary, MB fluorescence imaging is a major method for ureteral visualization in gynecological surgery, potentially reducing injury risk. However, its multiple adverse effects may interfere with surgery and recovery, necessitating thorough patient evaluation before use.

ICG is another common fluorophore extensively studied for ureteral imaging with good results. Sam Siddighi et al. [52]successfully visualized bilateral ureters in 10 pelvic surgery patients by injecting ICG via ureteral catheters. ICG combined with ureteral catheters provides clear imaging throughout surgery without postoperative complications, compensating for lack of tactile feedback and enhancing safety [53, 54]. Current research primarily involves colorectal surgery, showing 100% success rates and no adverse events with retrograde injection of 5 ml of 2.5 mg/ml ICG solution [53–62]. In gynecology, Raimondo D. et al. [63] performed laparoscopic deep infiltrating endometriosis (DIE) resection in 23 patients with ureteral involvement (31 ureters). IV ICG (0.25 mg/kg) visualized 28 ureters (90.3%). Five ureters appeared pale/ischemic under white light; 3 showed irregular fluorescence (ischemia) under NIR-ICG, while 2 showed no ischemia, avoiding stent placement. Delgadillo Chabolla et al. [64] using robotic-assisted vNOTES for 53 hysterectomies and endometriosis resections with retrograde ICG injection (12.5 mg), achieved 100% visualization. In 2024, Barba M [65]. reported a successful case of ureteral visualization with ICG during transvaginal uterosacral ligament suspension, confirmed by postoperative cystoscopy. Note: Fluorescence brightness isn’t directly dose-dependent. Although ICG can penetrate up to 10 mm of soft tissue, imaging quality varies with BMI (e.g., less bright in morbid obesity) [52, 55]. Also, retrograde ICG injection may cause unclear distal ureter visualization initially (34.4%), requiring time for full enhancement [56]. As an FDA-approved safe drug, ICG is widely used in human surgery. Animal studies often involve new material development, e.g., ICG-based NIR resin catheters successfully visualized ureters in 5 female pigs with stable imaging, indicating potential for future development [66]. Using ICG for ureteral mapping has a learning curve, but it is short and suitable for training [63].

Beyond MB and ICG, other dyes have shown good efficacy. IRDye 800 BK is one. Lacking FDA approval, recent experiments use animals or cadavers. In 2020, Mahdi Al-Taher performed laparoscopic and robotic surgeries on female pigs using IV IRDye 800 BK (0.15 mg/kg), achieving long-lasting visualization (from 5 min post-injection to surgery end) without significant intensity decay, facilitating ureter identification [14, 67]. Subsequent studies confirmed good, stable visualization at 0.15 mg/kg without adverse events [68–70].

ASP5354, in a study of 12 laparoscopic rectal surgeries using different IV doses, showed dose-dependent efficacy: 66.7% success at 0.1 mg, 83.3% at 1.0 mg, and 100% at 3.0 mg [12, 13]. Higher doses improve visualization but increase adverse event risk.

Other agents like fluorescent catheter KFL-4 [71], zwitterionic ZW800-1 [15], UL-766 [16], and UreterGlow [25] also show promising effects without reported adverse events. Except for ICG, these agents lack FDA approval and are still in development with limited human studies. However, increasing animal studies and clinical demand suggest more options will become available for different surgical scenarios (Table 3). 

**Table 3:** Summary table of ureter visualization methods

Dye/Tracer	Injection Method	Dosage	Imaging Time	Advantages	Disadvantages
MB	Intravenous	0.75−1.0 mg/kg	Begins 10 min post-injection, lasts 60–75 min	Long visualization time, penetration depth 3–5 mm, low cost	May cause decreased oxygen saturation, allergies, urine staining, limited penetration
ICG	Retrograde via ureteral catheter	2.5 mg/ml, 5 ml	Immediate visualization post-injection, lasts until end of surgery	High safety, no adverse reactions, clear visualization	Decreased fluorescence brightness in obese patients, potential delayed distal visualization
ICG	Intravenous	0.25 mg/kg	Visualizes within minutes post-injection	Allows assessment of ureteral blood flow, non-invasive	High abdominal background fluorescence, susceptible to liver uptake, low renal excretion
IRDye800BK	Intravenous	0.15 mg/kg (animal studies)	Visualizes 5 min post-injection, remains stable	Primarily renal excretion, stable fluorescence, strong penetration	Not FDA approved, limited human studies
ASP5354	Intravenous	1.0–3.0 mg	Dose-dependent, optimal at 3.0 mg	High fluorescence intensity, rapid renal clearance, almost complete excretion	Risk of dose-dependent adverse reactions, investigational stage
Fluorescent Catheter (e.g., KFL−4)	Fluorescent resin catheter	Fluorescent agent built into catheter	Continuous visualization after catheter placement	Stable visualization, enables intraoperative visualization all the time	Requires specialized catheter, high cost, not widely used
ZW800−1	Intravenous	Dose not specified (investigational)	Rapid visualization, good penetration depth	Low background fluorescence, rapid renal clearance	Safety not fully verified
UL−766	Intravenous	Effective at low doses	Rapid visualization, high fluorescence intensity	High renal clearance rate, high sensitivity	Insufficient toxicity studies, not commercially available

* *ICG* Indocyanine Green, *MB* Methylene Blue, ^99m^*Tc* Technetium-99m

## Limitations of current fluorescence imaging systems

Although many commercially available fluorescence imaging systems provide high‑quality images, and novel fluorophores are continuously emerging and being applied to an increasing number of surgical specialties, their operation remains relatively cumbersome in procedures requiring composite imaging. For example, in gynecologic malignancy surgery, lymphadenectomy is one of the standard procedures and serves as the primary basis for tumor pathological staging. Because the pelvic lymph nodes are in close proximity to the ureters, surgeons must constantly monitor the ureteral course when dissecting the pelvic lymph nodes — a stage at which the ureters are most vulnerable to injury. If MB and ICG are used to visualize the ureters and lymph nodes, respectively, the surgeon must switch back and forth between different fluorescence wavelengths intraoperatively to locate the two structures. This approach is not only operationally cumbersome but also prone to positioning errors. Although many other fluorophores are available, this issue remains critical. Consequently, how to achieve simultaneous intraoperative imaging of multiple fluorescence wavelengths and integrate them with white‑light images to obtain a clear, bright surgical field with precise localization has become a key technical challenge that major medical device manufacturers have been striving to address in recent years. 

## Progress in domestic fluorescence laparoscopy

The development of fluorescence endoscopy dates to the 1960s, initially using hematoporphyrin derivative (HPD), ICG, and 5-aminolevulinic acid (5-ALA) for tumor detection. In the 1980s, NIR/ICG fluorescence imaging was studied for angiography and liver function assessment. Stryker entered the endoscope market in 1981, later launching the world’s first fluorescence endoscope. In the early 2000s, several endoscopic fluorescence systems achieved commercial clinical use. In 2009, Novadaq’s first laparoscope introduced widespread clinical use of NIR fluorescence laparoscopy. In 2012, Novadaq launched the PINPOINT Gen-2 system, a market-first NIR imaging system still widely used. That same year, Olympus introduced a multi-functional autofluorescence imaging system allowing rapid switching between white light, autofluorescence, and narrow-band imaging (NBI). Stryker’s acquisition of Novadaq in 2017 solidified its leading position and introduced fluorescence endoscopy to China. Currently, Stryker leads the global fluorescence endoscope market (> 70% share) and is the largest foreign brand in China.

Domestically, fluorescence technology is often integrated with 4 K. Over 15 Chinese companies have approved 4 K+fluorescence products, including Guangdong OptoMedi, Mindray, New Imaging Vision, Kangji, and United Imaging Healthcare. Founded in 2013, OptoMedi is a major challenger to Stryker, capturing 30% of China’s fluorescence endoscope market in just 9 years. According to Frost & Sullivan, Stryker and OptoMedi now share the domestic market as the two major players.

Fluorescence technology continues to advance. At the November 2022 China International Import Expo, KARL STORZ introduced the globally unique IMAGE1 S Rubina system integrating 3D, 4 K, and NIR imaging, stirring the domestic market. Starting July 2023, within a month, three domestic companies launched four 3D/4K/NIR endoscopes approved for market: Mindray’s UX7 and UX5 series (China’s first certified 3D/4K/NIR product), Jin Hai Medical’s Endo Next True 4 K + 3D 4MOS fluorescence system, and OptoMedi’s Stellar 4 K Optical 3D Fluorescence Laparoscope System. In March 2024, OptoMedi achieved another breakthrough with the world’s first 4 K dual-fluorescence endoscopic camera system–the OptoMedi 214KM, enabling individual or combined ICG and MB fluorescence navigation. Under white‑light mode, structures are visualized in different colors — lymph nodes appear green (ICG) and the ureters blue (MB) — ensuring simple recognition and high accuracy. In gynecologic oncology procedures, the dual‑fluorescence mode allows surgeons to maximally reduce the likelihood of ureteral injury during pelvic lymphadenectomy.

## Discussion

Since the introduction of laparoscopic surgery in gynecologic malignancies, technological advances have driven concomitant improvements in surgical instruments, providing surgeons with considerable convenience and yielding positive feedback on clinical outcomes. Over the past decades, systematic pelvic lymphadenectomy appeared to be an inevitable component of surgical management for gynecologic malignancies. Pelvic lymphadenectomy, since its inception by Wertheim, has remained a core element of surgery for early‑stage cervical cancer; however, this procedure has inherent drawbacks. It prolongs operative time and increases the risks of vascular and nerve injury, lymphedema, ureteral injury, and other complications. Moreover, most early‑stage patients have no lymph node metastasis, and excessive resection may disrupt the anatomical basis of immune surveillance. SLN biopsy, by precisely identifying nodes at high risk of metastasis, can avoid extensive lymphadenectomy; nevertheless, comparative data on survival outcomes versus conventional surgery from large‑scale randomized controlled trials are lacking, which has precluded a consensus in clinical guidelines. The Chinese multicenter PHENIX trial [72] published in 2025 demonstrated that ICG‑guided SLN biopsy alone was non‑inferior to lymphadenectomy with respect to disease‑free survival in early‑stage cervical cancer patients, and was associated with a lower complication rate. Therefore, sufficient evidence currently supports the feasibility of SLN biopsy in cervical cancer surgery, whereas its role in endometrial cancer surgery remains under investigation.

**Fig. 1:**
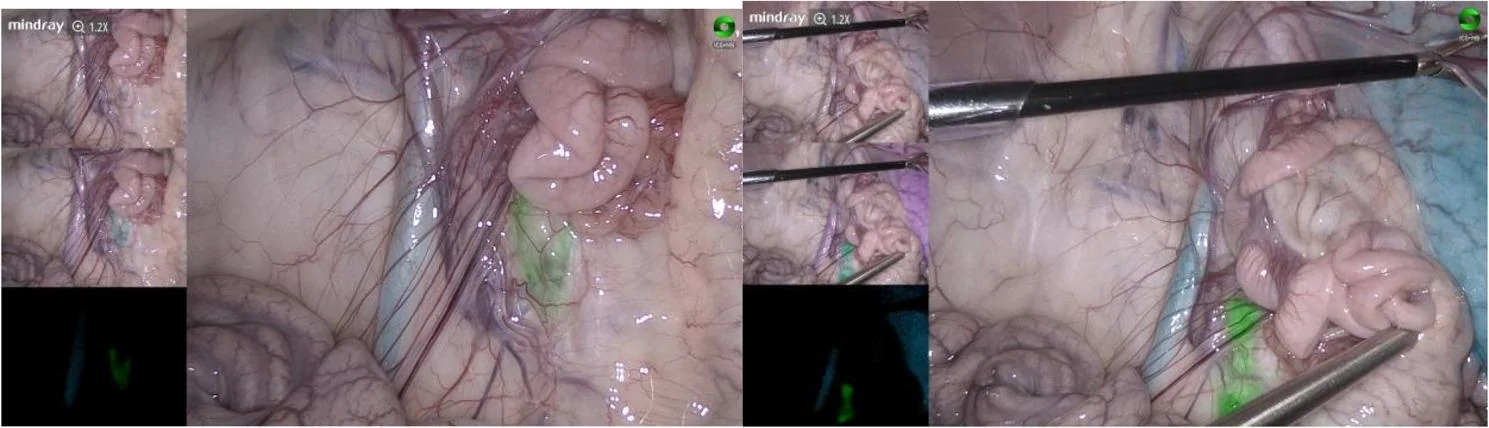
This shows ureteral and SLN mapping in a female pig, using intravenous injection of MB and cervical four‑quadrant injection of ICG. The two contrast agents were visualized simultaneously on a single screen, displayed in different colors through machine algorithm integration into the white‑light mode, providing a clear surgical field

**Fig. 2:**
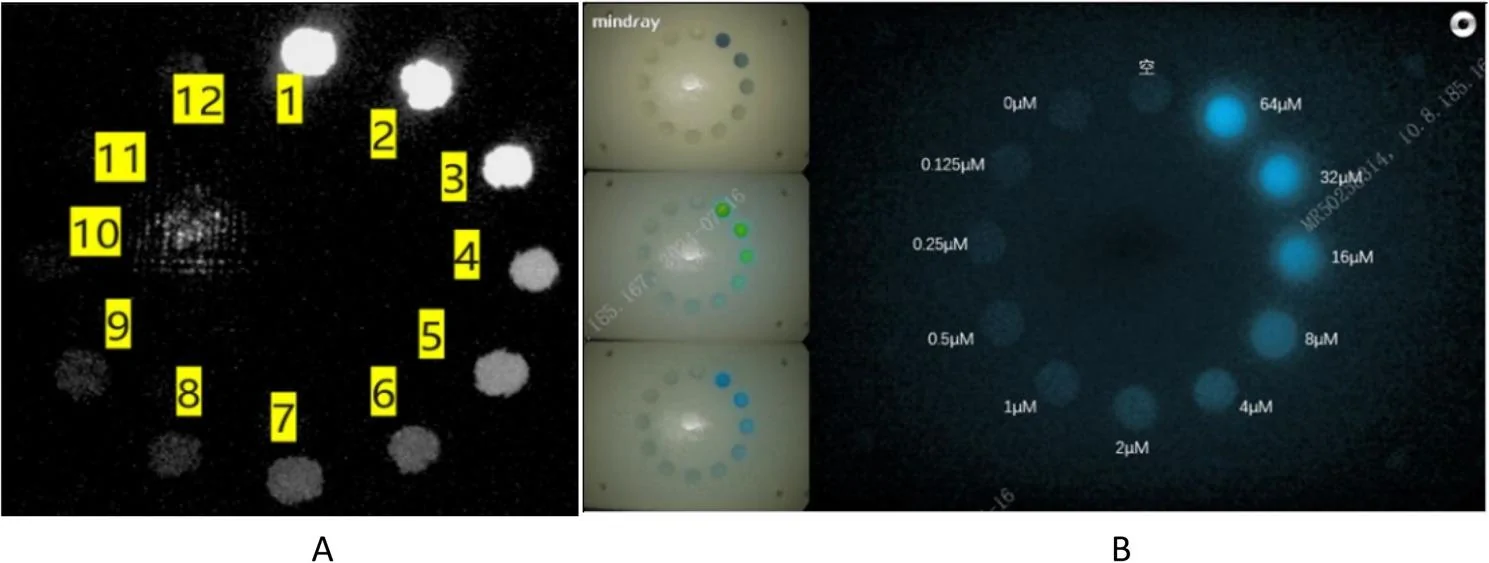
Evaluation of the dual‑fluorescence imaging system for visualizing ICG and MB across different concentrations. **A** Visualization performance of ICG at various concentrations under black-and-white fluorescence imaging. **B** The fluorescence performance of different concentrations of MB was evaluated in the dual‑fluorescence imaging system

Nevertheless, a serious adverse event was reported in one SLN mapping study: ureteral injury occurring during SLN biopsy and resection. The study attributed this to bleeding during resection, which compromised accurate localization of the retroperitoneal ureter when near‑infrared imaging was activated. Consequently, thermal injury to the ureter occurred; fortunately, it was detected early and intraoperative ureteral stenting was performed, with no long‑term sequelae for the patient. Had dual‑fluorescence imaging (intravenous MB combined with cervical ICG injection) been available intraoperatively, such injury might have been avoided. Our team conducted an animal experiment using a domestically produced dual‑fluorescence system, with intravenous MB at 0.025 mg/kg and four‑quadrant cervical ICG injection (Fig. 1). The imaging device enabled simultaneous visualization of the ureter and SLN on a single screen. Prior to this experiment, we tested the fluorescence performance of the dual‑fluorescence system for ICG and MB. When MB was diluted to the typical clinical concentration (1%), it exhibited bright blue color visible to the naked eye, but due to quenching, it failed to produce near‑infrared (NIR) fluorescence in porcine urine. However, when diluted to 16 µM in phosphate‑buffered saline (PBS), MB displayed moderate NIR fluorescence (Fig. 2A), with an excitation peak at 665 nm and an emission peak at 684 nm. Using the dual‑fluorescence imaging system, from top to bottom on the left side, we observed: a combined dual‑fluorescence image under white‑light mode, an image at excitation wavelength 700–800 nm (green fluorescence), and an image at 600–700 nm (blue fluorescence). Additionally, ICG at various concentrations showed different fluorescence intensities in grayscale fluorescence imaging (Fig. 2B).

Notably, the previous concern about intraoperative inability to distinguish ureteral fluorescence from SLN fluorescence may be alleviated with this device, as it can display MB as blue fluorescence and ICG as green fluorescence via the monitor’s machine algorithm, differentiating the two wavelengths and overlaying them with white‑light imaging, thereby providing a brighter surgical field during SLN resection. Of course, clinical studies are currently ongoing; if results from clinical trials become available, they may provide intraoperative decision algorithms and evidence to support clinical decision‑making in the future.

At present, the OptoMedi 214KM dual‑fluorescence endoscopic camera system has been approved for market after sufficient registered clinical validation and rigorous expert panel review. The integration of two fluorescence contrast technologies into a single system is not a simple superposition but a challenge to the enterprise’s high‑level integrated design capability and manufacturing standards. However, its relatively high cost has limited its clinical adoption. Another domestically produced dual‑fluorescence laparoscope, from Mindray Medical, also integrates dual fluorescence into one device; however, it has not yet been approved for market and commenced clinical trials in 2024.

Evidently, the choice of imaging modality should be individualized based on patient factors. For SLN mapping, ICG is widely adopted due to its lymphatic affinity and high signal‑to‑noise ratio. For ureteral imaging, MB enables non‑invasive visualization via intravenous injection but has limited penetration depth; retrograde ICG injection, though requiring catheterization, achieves nearly 100% visualization. The choice should be based on patient body mass index (BMI), surgical approach, and available equipment. Although MB effectively visualizes the ureter, its adverse effects (including risk of serotonin syndrome, interference with pulse oximetry, and blue discoloration) limit its widespread use. MB is contraindicated in patients taking SSRI antidepressants. When retrograde ICG injection or novel renally excreted dyes (e.g., ASP5354) are available, these alternatives may be safer. Additionally, CW 800‑CA is an FDA‑approved contrast agent that enables real‑time ureteral visualization after intravenous injection; however, due to limited clinical data, it currently has lower priority than MB in clinical practice. For patients with high BMI, agents with high tissue penetration, such as ICG or the fluorescent catheter KFL‑4 [71], should be selected. In early‑stage cervical or endometrial cancer surgery, MB for ureteral imaging is a reasonable choice. For advanced gynecologic tumor surgery, to achieve ureteral protection, the fluorescent catheter KFL‑4 or retrograde ICG imaging after ureteral stenting should be chosen; although this prolongs operative time, it enables combined tactile and visual ureteral protection.

## Clinical recommendations

### Endometriosis, early‑stage endometrial cancer, and early‑stage cervical cancer

Intravenous MB is recommended for ureteral imaging; in gynecologic oncology patients, it can be combined with ICG for SLN mapping, and a dual‑fluorescence laparoscopic system should be used for display.

### Severe pelvic adhesions and advanced gynecologic tumors

The fluorescent catheter KFL‑4 is recommended for ureteral imaging, or ureteral stenting with retrograde ICG imaging.

### Obese patients

Retrograde ureteral imaging with high‑penetration ICG is recommended.

## Data Availability

No new data were created or analyzed in this study. Data sharing is not applicable to this article.
